# Associations of Gut Microbiota and Fatty Metabolism With Immune Thrombocytopenia

**DOI:** 10.3389/fmed.2022.810612

**Published:** 2022-05-19

**Authors:** Xiaomin Yu, Qingyun Zheng, Yun He, Dandan Yu, Guolin Chang, Cheng Chen, Laixi Bi, Jia Lv, Misheng Zhao, Xiangyang Lin, Liqing Zhu

**Affiliations:** ^1^Key Laboratory of Clinical Laboratory Diagnosis and Translational Research of Zhejiang Province, Department of Clinical Laboratory, The First Affiliated Hospital of Wenzhou Medical University, Wenzhou, China; ^2^State Key Laboratory of Genetic Engineering, School of Life Sciences, Fudan University, Shanghai, China; ^3^Department of Hematopathology, The First Affiliated Hospital of Wenzhou Medical University, Wenzhou, China; ^4^Department of Pathology, The Second Affiliated Hospital of Wenzhou Medical University, Wenzhou, China; ^5^Department of Clinical Laboratory, Wenzhou People’s Hospital, Wenzhou, China

**Keywords:** immune thrombocytopenia, gut microbiota, fatty metabolism, 16S rRNA, cytokines

## Abstract

**Objective:**

To determine whether gut microbiota, fatty metabolism and cytokines were associated with immune thrombocytopenia (ITP).

**Methods:**

In total, 29 preliminarily diagnosed ITP patients and 33 healthy volunteers were enrolled. Fecal bacterial were analyzed based on 16S rRNA sequencing. Plasma cytokines and motabolites were analyzed using flow cytometry and liquid chromatography-mass spectrometry (LC-MS), respectively.

**Results:**

*Bacteroides*, *Phascolarctobacterium*, and *Lactobacillus* were enriched at the genus level in ITP patients, while *Ruminococcaceae UCG-002*, *Eubacterium coprostanoligeues*, *Megamonas, and Lachnospiraceae NC2004* were depleted. At the phylum level, the relative abundance of *Proteobacteria* and *Chloroflexi* increased in ITP patients, while *Firmicutes*, *Actinobacteria*, and the *Firmicutes/Bacteroidetes* ratio decreased. Plasma levels of 5-hydroxyeicosatetraenoic acid (5-HETE), 6-trans-12-epi-leukotriene B4 (6t,12e-LTB_4_), and resolvin D2 (RvD_2_) were upregulated, and stachydrine, dowicide A, dodecanoylcarnitine were downregulated in ITP patients. Furthermore, RvD_2_ is positively correlated with order *Bacteroidetes VC2.1 Bac22*, 5-HETE is positively correlated with genus *Azospirillum*, and 6t,12e-LTB_4_ is positively correlated with genus *Cupriavidus*. In addition, stachydrine is positively correlated with family *Planococcaceae*, dowicide A is positively correlated with class *MVP-15*, and dodecanoylcarnitine is positively correlated with order *WCHB1-41*. Plasma levels of interleukin-6 (IL-6) and tumor necrosis factor-α (TNF-α) were upregulated in ITP patients.

**Conclusion:**

Our study revealed a relationship between microbiota and fatty metabolism in ITP. Gut microbiota may participate in the pathogenesis of ITP through affecting cytokine secretion, interfering with fatty metabolism.

## Introduction

Immune thrombocytopenia (ITP) is characterized by low peripheral blood platelet count. It is an acquired autoimmune disorder which can lead to easy and excessive bruising and bleeding. The increased platelet clearance arises through diverse mechanisms, such as antiplatelet autoantibodies production, and T lymphocyte dysfunction. It was reported that receptors for the Fc fragment of IgG result in the removal of platelets coated with immunoglobulin (1) and the activation of phagocytosis in the reticuloendothelial system (2). In addition to B cell dependent autoimmune mechanisms, it is well established that cellular immune pathophysiology of ITP is associated with several T-cell abnormalities, including uncontrolled activation of T helper cells, deficiency of T regulatory cells, and increase of CD8 + T cell targeting megakaryocytes, etc. (2, 3). Another mechanism that contributes to the platelet clearance is platelet activation (4). In two pilot studies, it was reported that platelet activation markers, such as P-selectin, CD62 and CD11b expression, were significantly higher in ITP patients (5, 6). In the ITP mouse model, Fc-independent platelet activation was observed, which led to platelet clearance in the liver (7). This partially explained the fact that the risk of venous thromboembolism is twice higher in ITP patients than in healthy population (8). More recently, COVID-19 vaccines have been linked with serious vaccine-induced ITP (9), which is believed to be mediated by activated platelets (10, 11). However, the initial trigger of platelets activation has not yet to be determined.

Emerging data has demonstrated a strong association among platelet function, metabolites of gut microbiota, and lipid metabolism (12). Two gut microbiota derived-metabolites, trimethylamine N-oxide (13) and phenylacetylglutamine (14) were shown to directly contribute to platelet activation related phenotypes. It is worth noticing that the metabolite phenylacetylglutamine was discovered through untargeted metabolomics, a powerful and unbiased platform for discovery of pathways linked to diseases. Using the similar strategy, analysis of a cohort of 534 healthy adult Dutch volunteers provided evidence for the importance of intestinal microbiota in regulating platelet function (15). More than 20% metabolites were significantly associated with platelet activation markers and the majority involved lipids. In a more recent study, clinical findings with experimental data suggested that gut microbiota dysbiosis may be an important mechanism for the high platelet reactivity in patients treated with ticagrelor, a first-line drug for the treatment of acute ST elevation myocardial infarction (16). For example, the abundance of gut microbiota species *Methylbacillus*, *Sphingomonas*, and *Staphylococcus* was negatively correlated with serum salicylate, which was previously found to induce ITP and inhibit platelet production in megakaryocytes (17).

Platelet metabolism and reactivity critically depend on lipid composition (18). For example, arachidonic acid (AA), a polyunsaturated fatty acid constituting an important part of phospholipid-content in cell membranes, is associated with the enzyme-derived oxidized metabolites (19). It enters the cell, activates the platelet and exerts in platelet function and fate. Up to 25% of phospholipid fatty acids are AA in resting platelets, the concentration of which may influence normal cellular functions and development of platelet diseases (20). Other metabolites like prostacyclin (PGI2) and prostaglandin D2 (PGD2) also inhibit platelet activation (21, 22) while 12(S)-HETE produced by 12-LOX was found to impact the platelet function (23). Besides, it was observed that high-density lipoprotein (HDL) reduced platelet aggregation in response to a variety of agonists, while oxidized HDL exerted the opposite effect binding to a similar receptor (24). In general, specific lipid species play important roles in enhancing stability of the domains and stabilizing signaling events in platelets, which permit the feasibility of a systematic assessment on platelet-lipid analysis (18).

To date, the analysis of gut microbes and lipid metabolism in modulation of ITP-associated platelet function has not yet been reported. The present study should provide new insights in the pathogenesis mechanisms associated with gut microbiota, cytokine, fatty metabolism in ITP, and therefore could improve differential diagnosis and support further treatment decision, such as traditional Chinese medicine, for ITP patients.

## Materials and Methods

### Subjects and Sample Collection

Fecal and plasma samples were collected from patients with newly diagnosed ITP before systemic treatment at the First Affiliated Hospital of Wenzhou Medical University. None of the recruited patients and healthy volunteers presented any other autoimmune diseases or intestinal and metabolic diseases that could affect the gut microbiota. The protocol was approved by the Ethics Committee of Wenzhou Medical University (KY2021-048), and informed consent was obtained from all participants. ITP was diagnosed according to the criteria established by the American Society of Hematology (ASH), which identifies as an autoimmune disorder characterized by an isolated platelet count < 100×10^9^/L, with or without bleeding manifestations, in the absence of other causes or disorders that may be associated with thrombocytopenia (25). In total, 29 patients were enrolled in the present study. In addition, 33 volunteers were recruited after a routine physical examination. All healthy controls presented no gut problems such as chronic diarrhea, constipation, gas, heartburn, bloating (26), and none had taken any antibiotics or probiotics within 3 months of this study. Moreover, there were no significant differences between the two groups in terms of sex, age, smoking history, and alcohol or dietary intake.

### Cytokines Detected by Flow Cytometry

For cytokine analysis, venous blood (2 mL) was collected and centrifuged to obtain 200 μL of serum. Cytokines, including interleukin (IL)-2, IL-4, IL-6, IL-10, TNF-α, and interferon (IFN)-γ, were detected by employing a cytometric bead array human inflammation kit according to the manufacturer’s instructions (BD Parmingen, San Diego, CA, United States) and operated using AimPlex™ bead-based multiparametric flow cytometry (EPICS-Elite, Beckman-Coulter, United States). For determining the protein concentration, sample fluorescence signals were compared with those of a standard curve generated from serial dilutions of a known concentration of the analyte.

### 16S rRNA Gene Sequencing

#### DNA Extraction and Polymerase Chain Reaction Amplification

Genomic DNA was extracted based on the V4 hypervariable region of 16S rDNA using a fecal genomic DNA extraction kit (QIAGEN), with 0.8% agarose gel electrophoresis performed to separate DNA. Then, to amplify the bacterial 16S rRNA from diluted DNA extracts, we used template-specific sequences 515F (5′-GTGCCAGCMGCCGCGGTAA-3′) and 806R (5′GGACTACHVGGGTWTCTAAT-3′). Polymerase chain reaction (PCR) amplification was performed using a 25 μL reaction volume with the following composition: 1 × PCR buffer, 1.5 mM MgCl_2_, 0.4 μM dNTPs, 1.0 μL forward primer, 1.0 μL reverse primer, 0.5 U KOD-Plus-Neo enzyme (TOYOBO), and 10 ng template. The reaction was initiated at 94^°^C for 1 min, followed by 30 cycles of denaturation at 94^°^C for 20 s, annealing at 54^°^C for 30 s, and elongation at 72^°^C for 30 s, with a final extension step at 72^°^C for 5 min. Each sample was assessed in triplicate. PCR products were mixed with 1/6 volume 6 × loading buffer and detected by 2% agarose gel electrophoresis. These amplicons were recycled using QIAquick Gel Extraction Kit (QIAGEN), quantified using Qubit@ 2.0 Fluorometer (Thermo Scientific), and finally mixed equally. The library was prepared using TruSeq DNA PCR-Free Sample Prep Kit (FC-1213001/3003) (Illumina Inc., San Diego, CA). After quantification and detection, the sequencing reaction was conducted using the PE250 pattern of Hiseq Rapid SBS Kit v2 (FC-402-4023 500 Cycle) (Illumina Inc., San Diego, CA).

#### Microbial Diversity Analysis

After sequencing, high-quality sequences were filtered for subsequent analysis and assembled from raw data. Alpha diversity [Chao1, phylogenetic diversity (PD), Simpson, Shannon] and beta diversity (PCoA) were analyzed using Usearch and QIIME. Statistics and plotting were completed using R6, Python, and Java. Next, to determine the species composition and diversity, high-quality sequences were clustered with a cut-off of 97% similarity, resulting in a total of 6,327 operational taxonomic units (OTUs). For each OTU, the sequence that occurred most frequently was selected as the representative OTU sequence. Annotation analysis was performed using UCLUST and SILVA databases. Multiple sequence alignment was performed using PyNAST, with the phylogenetic tree constructed using FastTree. Samples were homogenized and resampled with the standard of the least amount.

### Metabolomics Analysis

#### Metabolite Extraction

After plasma samples were thawed on ice, 120 μL of precooled 50% methanol buffer was added to 20 μL of samples. Then, samples were vortexed for 1 min, incubated for 10 min at room temperature and stored overnight at −20^°^C. The mixture of metabolites was centrifuged at 4,000 × g for 20 min to obtain the supernatant. Subsequently, supernatants were transferred into new 96-well plates and stored at −80^°^C for subsequent liquid chromatography-mass spectrometry (LC-MS) analysis. For pooled quality control (QC) samples, 10 μL of each extraction mixture were combined and mixed.

#### Liquid Chromatography-Mass Spectrometry Analysis

All samples were analyzed using the TripleTOF 5600 Plus high-resolution tandem mass spectrometer (SCIEX, Warrington, United Kingdom), utilizing both positive and negative ion modes. Chromatographic separation was performed in an ultra-performance liquid chromatography (UPLC) system (SCIEX, United Kingdom) using an ACQUITY UPLC T3 column (100 mm × 2.1 mm, 1.8 μm; Waters, United Kingdom) for reversed-phase separation, with a constant column temperature of 35^°^C. The mobile phase consisted of solvent A (water, 0.1% formic acid) and solvent B (acetonitrile, 0.1% formic acid). The UPLC elution conditions were optimized and set as follows: flow rate, 0.4 mL/min; 0–0.5 min, 5% solvent B; 0.5–7 min, 5–100% solvent B; 7–8 min, 100% solvent B; 8–8.1 min, 100–5% solvent B; 8.1–10 min, 5% solvent. The TripleTOF 5600 Plus System was used to detect eluted metabolites. The curtain gas was set at 30 PSI, with ion source gases 1 and 2 at 60 PSI. The temperature of the interface heater was 650^°^C. The ion spray floating voltage was set at 5 kV for the positive ion mode and −4.5 kV for the negative ion mode.

MS data were acquired in Information Dependent Acquisition mode. The time-of-flight mass ranged between 60 and 1,200 Da. Survey scans were acquired over 0.15 s, and up to 12 product ion scans were collected if the 100 counts/s threshold was exceeded, with a 1 + charge state. The total cycle time was fixed at 0.56 s. Four-time bins were summed for each scan at an impulsator frequency of 11 kHz by monitoring the 40 GHz multichannel TDC detector with four-anode/channel detectors. Dynamic exclusion was performed for 4 s. During the entire acquisition period, the mass accuracy was calibrated for every 20 samples. In addition, a QC sample was analyzed every 10 samples to evaluate the LC-MS stability.

#### Database Searches and Bioinformatics Analysis

The acquired LC-MS data was pretreated using XCMS software (SCIEX). Raw data was converted into mzXML format and processed using the XCMS, CAMERA, and metaX toolboxes included in R software. Each ion was identified using comprehensive information regarding the retention time and m/z. The intensity of each peak was recorded, and a three-dimensional matrix containing arbitrarily assigned peak indices (retention time –m/z pairs), sample names (observations), and ion intensity information (variables) were generated. Then, the information was matched to in-house and public databases. The open-access databases, KEGG (Kyoto Encyclopedia of Genes and Genomes)^1,2^ and HMDB (Human Metabolome Database)^3^, were employed to annotate metabolites by matching the exact molecular mass data (m/z) to those from the database, within a threshold of 10 ppm. Peak intensity data was further pre-processed using metaX software. Features detected in 30% of samples were removed. Group datasets were normalized before analysis. Data normalization was performed for all samples using a probabilistic quotient normalization algorithm. Then, QC-robust spline batch correction was performed using QC samples. The *P*-value was analyzed using the Student’s *t*-test, then adjusted for multiple tests using a false discovery rate (Benjamini–Hochberg) to select different metabolites. Supervised partial least-squares discriminant analysis was performed using metaX to identify variables that differed between groups. The variable importance in projection was calculated, and a cut-off value of 1.0 was set to select important features.

### Statistics Analysis

The results were statistically analyzed using SPSS version 22.0 (IBM Corp., Armonk, NY, United States). Data are expressed as mean ± standard error of the mean. Significant differences in clinical characteristics and cytokine levels between healthy controls and ITP patients were analyzed by independent-sample *t*-test. A *P*-value of <0.05 was considered statistically significant. We used Spearman correlation analysis to filter differential metabolites and gut microbiota.

## Results

### Demographic, Clinical, and Metabolite Parameters of Study Subjects

The present study recruited 62 subjects, including 29 ITP patients (13 men, 16 women) and 33 healthy controls (17 men, 16 women) (Table 1). No statistically significant difference was observed between the patient group and control group in terms of gender (male: 45% vs. male: 52%, *P* = 0.599) and age (39.50 ± 15.98 vs. 39.60 ± 15.87, *P* = 0.989) (Table 1). Serum levels of total protein (TP), alanine aminotransferase (ALT), alkaline phosphatase (ALP), gamma-glutamyl transpeptidase (GGT), glucose (GLU), urea, total cholesterol (TC), total triglyceride (TG), and low-density lipoprotein (LDL) were similar between patients and healthy controls. Levels of total bilirubin (TB, 9.93 ± 4.17 vs. 13.11 ± 4.33, *P* = 0.007), indirect bilirubin (IB, 5.86 ± 2.66 vs. 10.37 ± 3.67, *P*<0.001), albumin (ALB, 40.78 ± 4.94 vs. 45.21 ± 2.62, *P*<0.001), creatinine (Crea, 58.62 ± 16.92 vs. 69.93 ± 15.70, *P* = 0.012), uric acid (UA, 287.24 ± 87.61 vs. 345.37 ± 91.31, *P* = 0.018), and high-density lipoprotein (HDL, 1.11 ± 0.35 vs. 1.34 ± 0.27, *P* = 0.011) were lower in ITP patients compared to healthy controls (Table 1). In addition, levels of direct bilirubin (DB, 4.07 ± 1.85 vs. 2.74 ± 0.90, *P* = 0.001), globulin (GLB, 32.67 ± 6.85 vs. 29.23 ± 2.48, *P* = 0.016), and aspartate aminotransferase (AST, 21.57 ± 5.89 vs. 18.33 ± 3.28, *P* = 0.015) were higher in ITP patients than healthy controls (Table 1).

**TABLE 1:** Demographic, clinical and metabolite characteristics of the study participants.

Characteristic	ITP (*n* = 29)	HC (*n* = 33)	*P*-value
Sex (male)	13 (45%)	17 (52%)	0.599
Age (year)	39.50 ± 15.98	39.60 ± 15.87	0.989
TB (μmol/L)	9.93 ± 4.17	13.11 ± 4.33	0.007
DB (μmol/L)	4.07 ± 1.85	2.74 ± 0.90	0.001
IB (μmol/L)	5.86 ± 2.66	10.37 ± 3.67	<0.001
TP (g/L)	73.45 ± 5.18	74.43 ± 3.66	0.420
ALB (g/L)	40.78 ± 4.94	45.21 ± 2.62	<0.001
GLB (g/L)	32.67 ± 6.85	29.23 ± 2.48	0.016
ALT (U/L)	22.86 ± 15.24	19.52 ± 10.39	0.349
AST (U/L)	21.57 ± 5.89	18.33 ± 3.28	0.015
ALP (U/L)	71.59 ± 25.94	65.48 ± 18.05	0.315
GGT (U/L)	25.18 ± 16.59	21.22 ± 11.40	0.309
GLU (mmol/L)	6.06 ± 1.71	5.42 ± 0.89	0.088
Urea (mg/dl)	5.30 ± 1.36	4.69 ± 1.33	0.094
Crea (μmol/L)	58.62 ± 16.92	69.93 ± 15.70	0.012
UA (μmol/L)	287.24 ± 87.61	345.37 ± 91.31	0.018
TC (mmol/L)	4.51 ± 1.16	4.80 ± 0.92	0.316
TG (mmol/L)	1.33 ± 0.76	1.37 ± 0.60	0.822
HDL (mmol/L)	1.11 ± 0.35	1.34 ± 0.27	0.011
LDL (mmol/L)	2.63 ± 0.77	2.72 ± 0.71	0.654

*TB, total bilirubin; DB, direct bilirubin; IB, indirect bilirubin; TP, total protein; AlB, albumin; GLB, globulin; ALT, alanine aminotransferase; AST, aspartate aminotransferase; ALP, alkaline phosphatase; GGT, gamma-glutamyl transpeptidase; GLU, glucose; UA, uric acid; TC, total cholesterol; TG, total triglycerides; HDL, high density lipoprotein; LDL, low density lipoprotein.*

### Structure and Composition of Gut Microbiome in Immune Thrombocytopenia

**FIGURE 1:**
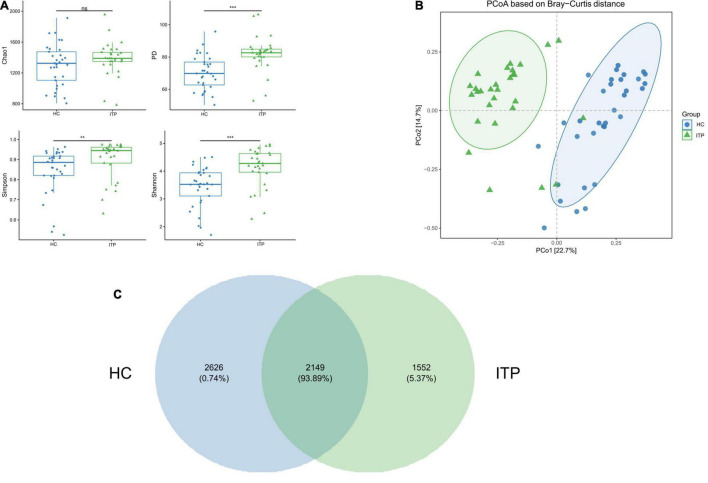
Significant differences of gut microbial community between ITP patients and healthy controls. **(A)** The alpha diversity indexes include Chao 1, PD whole tree, Simpson, and Shannon. There are significant differences in PD whole tree, Simpson, and Shannon, except for Chao1. **(B)** PCoA is a convenient method to observe the degree of difference and the rule of variation among samples. Each dot represents a sample. The more similar the microbial community composition, the closer the dots. The plot shows a distinct difference between ITP patients and healthy controls. **(C)** A Venn plot is used to demonstrate the bacterial microbiota structure, assessing which species are common and unique among groups or samples. **p < 0.01, ***p < 0.001.

Alpha and beta diversities were calculated to evaluate the structural differences of gut microbial community between ITP patients and healthy controls. Chao 1 is a measure of total richness and is particularly useful owing to a valid variance, which can be employed to calculate confidence intervals. PD whole tree reflects the sum of all branch-lengths on the constructed phylogenetic tree from all taxa. Simpson considers both species abundance (Richness) and uniformity (Evenness). Shannon reflects species numbers and evenness of species abundance. As shown in Figure 1A, PD whole tree, Simpson, and Shannon had a significant difference between two groups, which represent the richness and diversity of gut microbiome community. The beta diversity in Figure 1B was also showed differences according to the principal coordinates analysis (PCoA). A total of 2,149 OTUs were found to be shared between ITP patients and healthy controls, accounting for a similarity level of more than 93% (Figure 1C).

### Immune Thrombocytopenia-Associated Microbiome Changes

**FIGURE 2:**
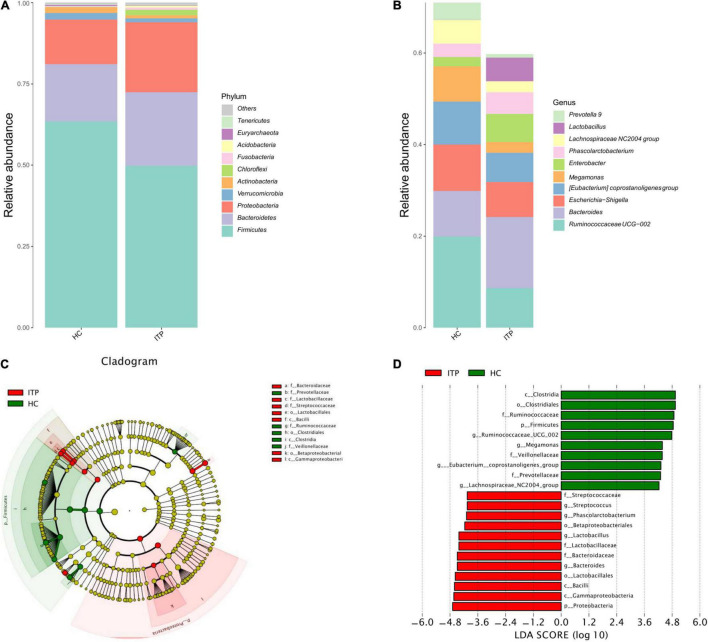
Differences of gut microbiota between ITP patients and healthy controls at phylum and genus level, respectively. **(A)** Relative abundance of bacterial microbiota in two groups at phylum level. **(B)** Relative abundance of bacterial microbiota in two groups at genus level. **(C)** The cladogram is constructed according to the hierarchical structure of classified data. **(D)** A linear discriminant analysis effect size (LEfSe) between ITP patients (ITP, green) and healthy controls (HC, red) revealed taxonomic abundances between groups (LDA > 4.0).

The top 10 ITP-associated bacterium at phylum level were shown in Figure 2A. *Proteobacteria* (*P* = 0.002), *Chloroflexi* (*P* < 0.001) were significantly high in ITP patients, while *Firmicutes* (*P* < 0.001), *Actinobacteria* (*P* < 0.001) were lower compared to healthy controls. Correspondingly, the *Firmicutes/Bacteroidetes* ratio was decreased. Next, we explored ITP-associated microbiome changes at genus level. As shown in Figure 2B, *Bacteroides* (*P* = 0.020), *Phascolarctobacterium* (*P* = 0.008), and *Lactobacillus* (*P* < 0.001) were significant high in ITP patients. While the *Ruminococcaceae UCG-002* (*P* < 0.001), *Eubacterium coprostanoligeues* (*P* = 0.011), *Megamonas* (*P* < 0.001), and *Lachnospiraceae NC2004* (*P* = 0.002) were significantly decreased.

### Significant Differences in Gut Microbial Taxa Between Immune Thrombocytopenia Patients and Healthy Controls

The cladogram was used for discovering the high-dimensional biomarkers that substantially contributed to the differences between groups. The main taxonomic groups of ITP patients and healthy controls were highlighted, respectively. Accordingly, *Lactobacillaceae* (family) and *Streptococcaceae* (family) belong to *Lactobacillales* (order), which belong to *Bacilli* (class) is highlighted (Figure 2C).

Next, we compared taxonomic abundances between ITP patients and healthy controls by employing linear discriminant analysis effect size (LEfSe), which was used to quantitatively analyze the important species that significantly differed among groups. The linear discriminant analysis (LDA) score correlates taxa abundancy which have the potential to be a biomarker. We listed those taxa which were meeting a significant LDA threshold more than 4 (LDA score > 4.0, *P* < 0.05). In this study, LEfSe analysis revealed more abundant *Proteobacteria* in ITP patients at phylum level, and more abundant *Bacteroides*, *Lactobacillus*, *Phascolarctobacterium*, and *Streptococcus* at genus level (Figure 2D).

### Differential Metabolite Expression

**FIGURE 3:**
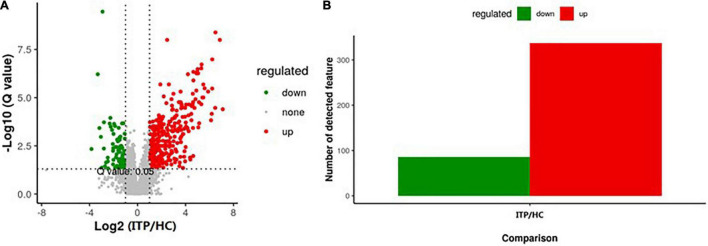
Alterations of metabolites of gut microbiota in ITP patients. **(A)** The vertical lines corresponded to a 2.0-fold change (up and down). The horizontal line represented a *P*-value of less than 0.05. The red and green dots represented upregulation and downregulation of microbiota, respectively. **(B)** Overall, 337 metabolites were significantly upregulated, while 86 were significantly downregulated.

Next, we explored the alteration of metabolites from gut microbiota in ITP. Specific diversity of metabolites was found between ITP patients and healthy controls. The metabolite expression levels were identified by volcano plot. As shown in Figure 3A, totally 423 metabolites which had a fold change more than 2.0 (*P* < 0.05) were identified from 4,786 metabolites. Among them, 337 were upregulated and 86 were downregulated (Figure 3B).

### Changes in Serum Cytokine Levels

**FIGURE 4:**
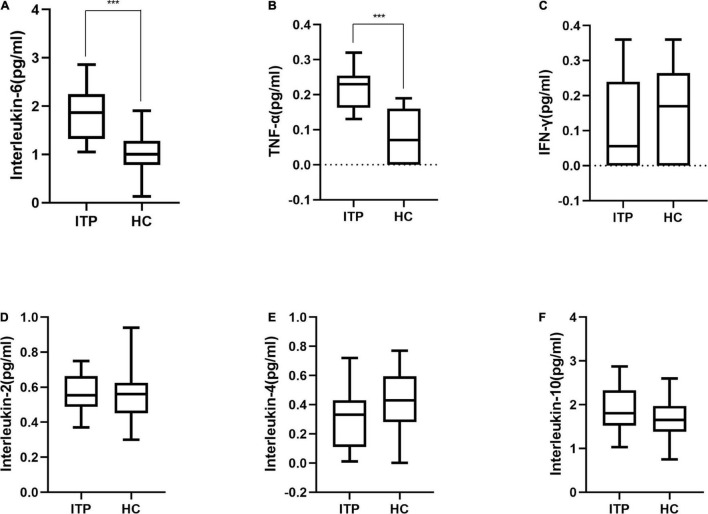
Box plot of cytokines in ITP patients and healthy controls. **(A)** Interleukin-6, **(B)** TNF-α, **(C)** IFN-γ, **(D)** Interleukin-2, **(E)** Interleukin-4, **(F)** Interleukin-10. These cytokines were all detected by flow cytometry. ***p < 0.001.

Furthermore, serum IL-2, IL-6, IL-4, IL-10, TNF-α, and IFN-γ were detected to reflect the immune response of ITP patients which were shown in Figure 4. IL-6 and TNF-α were significant higher in ITP patients compared to the healthy controls (*P* < 0.001, *P* < 0.001, respectively). While IL-2, IL-4, IL-10, and IFN-γ had no significant difference between two groups. However, a trend of down regulation of IL-4 and IFN-γ were observed in ITP patients, even they failed to reach a statistical significance.

### Potential of Metabolic Parameters as a Possible Indicator of Immune Thrombocytopenia

We then investigated whether the metabolic pathway could help predict the clinical prognosis of ITP. The PCA plot showed metabolites of ITP patients are different from healthy controls. Univariate analysis, fold change (Ratio ≥ 2 or ≤ 1/2), *t*-test (*P* value<0.05), and Variable Important for the Projection (VIP ≥ 1) were obtained by multivariate analysis, which were employed to filter the representative metabolic parameters. Compared with healthy controls, fatty acyls compounds including 5-HETE, RvD_2_, and 6t,12e-LTB_4_ were significantly upregulated. While stachydrine, dowicide and dodecanoylcarnitine were significantly downregulated. Hence, we speculated that lipid metabolism dysfunction might be occurred in the pathogenesis of ITP (Table 2).

**TABLE 2:** The metabolites were screened by some parameters.

Metabolite	MZ	RT	Ratio	*P*-value	VIP	Regulated	Class
Resolvin D2	375.22	3.42	137.23	<0.05	4.47	Up	Fatty acyls
5S-Hydroxy-6E,8Z,11Z,14Z-eicosatetraenoic acid	319.23	5.16	115.82	<0.05	4.63	Up	Fatty acyls
6-trans-12-epi-Leukotriene B4	335.22	3.96	69.88	<0.05	4.15	Up	Fatty acyls
1-Methylene-5.alpha.-androstan-3.alpha.-ol-17-one	325.21	5.00	33.39	<0.05	3.50	Up	Sterol lipids
Cinncassiol E	399.21	3.42	28.44	<0.05	3.66	Up	Prenol lipids
Octanoylcarnitine	288.22	3.00	0.27	<0.05	1.87	Down	Fatty acyls
Decanoyl-L-carnitine	316.25	3.22	0.25	<0.05	2.38	Down	Fatty acyls
Dodecanoylcarnitine	344.28	3.42	0.21	<0.05	2.53	Down	Fatty acyls
Dowicide A	237.05	0.84	0.13	<0.05	3.14	Down	Benzene and substituted derivatives
Stachydrine	144.10	0.89	0.12	<0.05	2.75	Down	Carboxylic acids and derivatives

*MZ, mass charge ratio; RT, retention time; Ratio, fold change; VIP, variable importance for projection.*

### Correlation Between Differential Metabolites and Gut Microbiota

**FIGURE 5:**
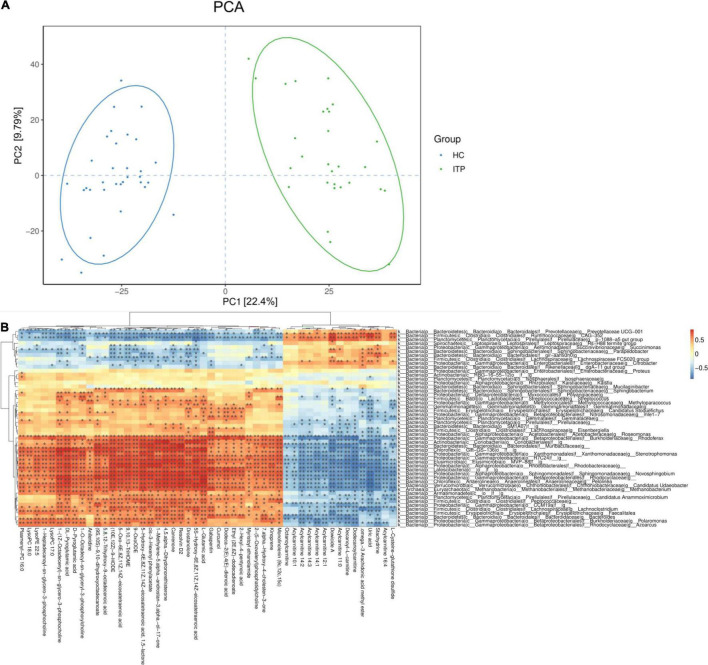
Relationship between serum metabolites and gut microbiota in ITP. **(A)** The PCA of ITP patients and healthy controls. **(B)** Heatmap of gut microbiota and metabolites. This heatmap shows a data matrix, where colored areas provided an overview of numeric differences.

In present study, interactions between gut microbiota and metabolites were investigated through metabolic and 16S sequencing analyses. Spearman correlation was performed to filter differential metabolites and gut microbiota. The Spearman’s rank correlation coefficient was showed as heatmaps of Figure 5. It was showed that RvD_2_, 5-HETE, 6t,12e-LTB_4_, stachydrine, dowicide A and dodecanoylcarnitineis were highly correlated with order *Bacteroidetes VC2.1 Bac22* (*r* = 0.91, *P* < 0.05), genus Azospirillum (*r* = 0.91, *P* < 0.05), genus Cupriavidus (*r* = 0.90, *P* < 0.05), family *Planococcaceae* (*r* = 0.88, *P* < 0.05), class *MVP-15* (*r* = 0.90, *P* < 0.05), order *WCHB1-41* (*r* = 0.85, *P* < 0.05), respectively (Table 3).

**TABLE 3:** The correlation of the differential metabolites and differential gut microbiota.

Metabolite	Microflora	*Rho*-value	*P*-value
Resolvin D2	k__Bacteria| p__Bacteroidetes| c__Bacteroidia| o__Bacteroidetes VC2.1 Bac22| f__| g_	0.91	< 0.05
5S-Hydroxy-6E,8Z,11Z,14Z-eicosatetraenoic acid	k__Bacteria| p__Proteobacteria| c__Alphaproteobacteria| o__Azospirillales| f__Azospirillaceae| g__Azospirillum	0.91	< 0.05
6-trans-12-epi-Leukotriene B4	k__Bacteria| p__Proteobacteria| c__Gammaproteobacteria| o__Betaproteobacteriales| f__Burkholderiaceae| g__Cupriavidus	0.90	< 0.05
1-Methylene-5.alpha.-androstan-3.alpha.-ol-17-one	k__Bacteria| p__Proteobacteria| c__Alphaproteobacteria| o__Azospirillales| f__Azospirillaceae| g__Azospirillum	0.89	< 0.05
Cinncassiol E	k__Bacteria| p__Bacteroidetes| c__Bacteroidia| o__Bacteroidetes VC2.1 Bac22| f__| g__	00.91	< 0.05
Octanoylcarnitine	k__Bacteria| p__Kiritimatiellaeota| c__Kiritimatiellae| o__WCHB1-41| f__| g__	0.77	< 0.05
Decanoyl-L-carnitine	k__Bacteria| p__Proteobacteria| c__Gammaproteobacteria| o__Aeromonadales| f__Succinivibrionaceae| g__Succinivibrionaceae UCG-002	0.83	< 0.05
Dodecanoylcarnitine	k__Bacteria| p__Kiritimatiellaeota| c__Kiritimatiellae| o__WCHB1-41| f__| g__	0.85	< 0.05
Dowicide A	k__Bacteria| p__Spirochaetes| c__MVP-15| o__| f__| g__	0.90	< 0.05
Stachydrine	k__Bacteria| p__Firmicutes| c__Bacilli| o__Bacillales| f__Planococcaceae| g__	0.88	< 0.05

## Discussion

Herein, we investigated the relationship among the gut microbiome, serum metabolism, cytokines in both the healthy people (*n* = 33) and ITP patients (*n* = 29), an autoimmune disorder. In addition to genetic and environmental triggers, accumulating evidence has suggested gut microbiota as another important causative element influencing the development of autoimmunity (27) as well as hematologic diseases (28). For example, there was evidence showing that gut bacterium contribute to type 1 diabetes development probably through translocation into pancreatic lymph nodes (28). For another example, inflammatory bowel disease with intestinal inflammation was reported to result in thrombocytopenia (29) and treatment led to recovery of the platelet number (30) indicating the link between hematology and gut microbiome. Indeed, Malnick et al. reported a case, in which the patient underwent fecal microbial transplantation twice from the same donor (31). Each time the patient developed transient ITP, with the platelet level decreasing and antiplatelet antibodies increasing rapidly after transplantation. These results, especially the recurrence of ITP after the second transplantation, strongly indicated that the gut microbiota is associated with the pathogenesis of ITP. However, the causality has not yet been established, with some arguments that the change of gut microbiome is only an outcome of disease progression rather than an initiator or exacerbator of disease.

Our results revealed that gut microbiota (diversity, richness, evenness, and PCA plot) and metabolites of fatty acids in ITP patients significantly differed from those in normal controls. Specifically, *Bacteroides*, *Phascolarctobacterium* and *Lactobacillus* were increased at the genus level, while *Ruminococcaceae UCG-002*, *Eubacterium coprostanoligeues*, *Megamonas* and *Lachnospiraceae NC2004* were decreased. At the phylum level, the relative abundance of *Proteobacteria* and *Chloroflexi* was increased in ITP patients, while *Firmicutes*, *Actinobacteria* and the *Firmicutes/Bacteroidetes* ratio were decreased. It was reported that *Firmicutes* (32) and *Firmicutes/Bacteroidetes* ratio (33) were associated with Th17-Tregs trans-differentiation, which is regulated by IL-6 (34). Interestingly, we found that IL-6 levels were significantly elevated in ITP patients when compared with healthy controls, which is consistent to the other reports showing that IL-6 upregulation is related to autoimmune diseases, such as rheumatoid arthritis, multiple sclerosis, and asthma (35). TNF-α is another cytokine that correlates with Treg frequencies in chronic ITP (36) and its secretion is stimulated by *Bacteroides* (37). It was evident that fecal microbiota transplantation reduced TNF-α signaling (38). Our results also showed that the *Bacteroides* composition and TNF-α level were elevated in ITP patients. For the fatty metabolites, those that were upregulated in ITP patients included RvD_2_, eicosatetraenoic acids, Monolinolein, and phosphatidylcholine, all of which can be regulated by gut microbiota (39). Interestingly, RvD_2_ was recently shown to reduce platelet activation and preserve platelet function (40). Eicosatetraenoic acid, also named arachidonic acid, is considered to regulate platelet homeostasis (41). In addition, eicosatetraenoic acids (41), Monolinolein (42), phosphatidylcholine (43), are all proposed to be the key compounds contributing to platelet activation. Nevertheless, at this moment, our understanding is still at its relative infancy regarding the exact roles of gut microbiota and fatty metabolism in the development of ITP. Apparently, this requires additional and longer-term studies in the future.

In summary, we attempt to obtain a comprehensive insight of altered gut microbiota and fatty metabolism in ITP patients. Our study may provide information for the treatment of ITP. Indeed, ITP is considered as purpura disease and differentiated as spleen deficiency in the TCMs. Nowadays in China, Many TCM drugs are routinely used to treat ITP *via* tonifying the spleen and qi, which may reduce the frequency and severity of bleeding (44) (Table 4). Meanwhile, TCM treatments were proven to be associated with changes in gut microbiota (61), and serum metabolic (62). Our data along with other literatures suggested that changes of gut microbiota in ITP patients may increase the toxicity of specific TCM drugs. For example, *Bacteroides* metabolizes geniposide to genipin with stronger hepatic toxic effect (45) and thus, geniposide-containing TCM formulations should be cautiously used to treat ITP. Further studies are needed with larger number of patients and animal experiments to establish the causality among gut microbiota, fatty metabolism and ITP.

**TABLE 4:** Roles of gut microbiota on toxicity of TCMs.

Gut microbiota	Specific mechanism	Influence on toxicity	Possibly affected TCM formulae/herbs	References
*Bacteroidetes*	Metabolizes geniposide to genipin with stronger hepatic toxic effect.	Increase	Qing-Kai-Ling Injection, Huang-Lian-Jiee-Dwu Tang, In-Chern-Hau Tang, Gardenia jasminoides Elli, Eucommia ulmoides Oliv., Rehmannia glutinosa Libosch., and Achyranthes bidentata Bl	(45)
	Metabolizes phorbol to isophorbol that induces ventricular fibrillation.	Increase	Croton tiglium, and thymelaeaceae	(46, 47)
	Metabolizes ginsenoside Rg3 to protopanaxadiol that exhibited the most potent cytotoxicity.	Increase	Panax ginseng	(48)
	Metabolizes rhein to rheinanthrone that is far more pronounced in laxative effect.	Increase	Yin-Chen-Hao Tang, Da-Cheng-Qi Tang, Rheum palmatum, and Duhaldea nervosa	(49)
	Metabolizes shikonin to its derivatives that are thought to have strong cytotoxicity	Increase	Lithospermum erythrorhizon, Arnebia euchroma, and Arnebia guttata	(50)
*Lactobacillus*	Transforms ginsenoside Rc into ginsenosides 20(S)-Rg_2_ and Rd, induces nephrotoxicity	Increase	Du-Shen-Tang	(51, 52)
	Ketonizes ginsenoside F1 and CK to new ketonized compounds with more potent inhibitory effects against mushroom tyrosinase	Increase	Panax ginseng	(53)
	Intervenes the tryptophan metabolism and causes diarrhea		Folium sennae	(54)
*Ruminococcus*	Enhances production of butyrate and G-protein-coupled receptors (GPRs) to support indigo naturalis to protect gut barrier	Decrease	Realgar-Indigo Naturalis	(55)
	Produces short chain fatty acids to assist schisandra chinensis to improve the gut micro-ecology	Decrease	Shegan-Mahuang Tang, Shengmai San, Fuzheng Huayu formula, Jian-Gan-Bao, CKBM, Shuang-Di-Shou-Zhen Tablets, Yangfei Huoxue Decoction, YiQiFuMai lyophilized injection powder, Wei Kang Su, Bakumijiogan	(56)
	Plays a crucial role in host energy utilization to promote Sophora flavescens EtOAc extract to rectify the abnormal energy metabolism	Decrease	Sophora flavescens, Qu-Yu-Jie-Du Decoction, San Wu Huangqin Decoction, ASHMI	(57)
	Increases short chain fatty acid level to support Gegen Qinlian Decoction to repair intestinal mucosa, ameliorates permeability, and alleviates diarrhea	Decrease	Gegen Qinlian Decoction	(58)
*Eubacterium*	Utilize lysine and arginine to produce acetate and butyrate to help Scutellariae radix and Coptidis rhizome to ameliorate the glycolipid metabolism	Decrease	Scutellariae radix, Coptidis rhizome, Huanglian Jiedu Decoction, Gegen Qinlian Decoction, San-Huang-Xie-Xin-Tang, Sanhuang-Siwu-Tang	(59)
	Produces butyrate to assist Xiexin tang to enhance epithelial barrier function, improve gut permeability, inhibit the inflammation and then exert systemically hypoglycemic activities	Decrease	Xiexin Tang	(60)

## Conclusion

Over the years, several researchers have explored the relationship between gut microbiota, metabolites, traditional Chinese medicine and autoimmune diseases. Advances in sequencing technology have made it possible to further explore the role of gut microbiota in autoimmune diseases. Our research revealed that gut microbiota and metabolites are associated with the pathogenesis of ITP and the gut microbiota might influence the metabolism of TCMs in ITP patients. Further experiments are required to determine the mechanisms of microbial species, the pathway of metabolome, the interaction between the gut microbiota and TCMs, and the pathogenesis of ITP.

## Data Availability Statement

The data presented in the study are deposited in NCBI Sequence Read Archive, accession number PRJNA816310.

## Ethics Statement

The studies involving human participants were reviewed and approved by the Ethics Committee in Clinical Research (ECCR) of the First Affiliated Hospital of Wenzhou Medical University. The patients/participants provided their written informed consent to participate in this study.

## Author Contributions

All authors listed have made a substantial, direct, and intellectual contribution to the work, and approved it for publication.

## Conflict of Interest

The authors declare that the research was conducted in the absence of any commercial or financial relationships that could be construed as a potential conflict of interest.

## Publisher’s Note

All claims expressed in this article are solely those of the authors and do not necessarily represent those of their affiliated organizations, or those of the publisher, the editors and the reviewers. Any product that may be evaluated in this article, or claim that may be made by its manufacturer, is not guaranteed or endorsed by the publisher.

## Footnotes

1
http://www.kegg.jp

2
http://www.genome.jp/kegg/

3
http://www.hmdb.ca
